# Biocontrol Efficacy of *Trichoderma* sp. THR3 Against *Sclerotium rolfsii* SCO1 Causing Damping-Off in Durian Seedlings

**DOI:** 10.3390/plants15213454

**Published:** 2026-11-05

**Authors:** Titi Thongkamngam, Sukritta Anutrakunchai, Bancha Wiangsamut

**Affiliations:** 1 Department of Plant Production Technology and Landscape, Faculty of Agro-Industrial Technology, Rajamangala University of Technology Tawan-Ok, Chanthaburi Campus, Chanthaburi 22210, Thailand; titi_th@rmutto.ac.th (T.T.); sukritta_an@rmutto.ac.th (S.A.); 2 Department of Agricultural Technology, Faculty of Agro-Industrial Technology, Rajamangala University of Technology Tawan-Ok, Chanthaburi Campus, Chanthaburi 22210, Thailand

**Keywords:** biological control, damping-off, durian rootstock, nursery disease, soil-borne pathogen

## Abstract

Damping-off disease threatens durian seedling production and can facilitate pathogen dissemination during transplanting. This study evaluated the biocontrol potential of *Trichoderma* sp. THR3 against *Sclerotium rolfsii* associated with damping-off of durian seedlings. Three isolates, SCO1, SCO2, and SCO3, were obtained from symptomatic seedlings and identified using morphological characteristics and internal transcribed spacer sequence analysis. In pathogenicity tests conducted on two-month-old durian seedlings, SCO1 was the most aggressive isolate and produced 100% disease incidence. In dual culture, *Trichoderma* sp. THR3 inhibited the mycelial growth of SCO1 by 88.7% at 7 days after inoculation. In the in planta experiment using one-year-old grafted durian plants, treatment with *Trichoderma* sp. THR3 resulted in a 50% numerical reduction in final disease severity caused by *S. rolfsii* SCO1 relative to the pathogen-inoculated control at 9 days after inoculation (DAI). Disease incidence was numerically lower in THR3-treated plants (60%) than in the pathogen-inoculated control (100%); however, this difference was not statistically significant (*p* > 0.05). These findings indicate the potential of *Trichoderma* sp. THR3 as a candidate biological control agent against *S. rolfsii* SCO1 in grafted durian nursery plants, although further replicated experiments are needed to confirm its efficacy under nursery and field conditions.

## 1. Introduction

Durian (*Durio zibethinus* L.) is one of Thailand’s most important economic fruit crops, widely cultivated for both domestic consumption and export [1]. Major export destinations include China; Vietnam; Hong Kong, China; Taiwan, China; the United States; and India. In 2023, durian-producing areas in Thailand covered approximately 169,211.84 hectares (ha), yielding around 1,476,174 tons of fruit, with an average yield of 8.724 t/ha. This marked an increase from 2022, when the production area totaled 156,607.84 ha, the overall yield was 1,335,728 tons, and the average yield was 8.529 t/ha [2]. Durian is primarily cultivated in the eastern region of Thailand, particularly in Rayong, Chanthaburi, and Trat provinces [3]. In these provinces, durian cultivation continues to expand, resulting in increasingly favorable environmental conditions for the occurrence and spread of plant diseases. As a result, the quantity of durian available for market distribution tends to decline, while consumer demand for durian continues to rise. Consequently, durian diseases have become a major challenge in meeting market demand. Various diseases currently cause significant losses to durian growers, including root and stem rot, pink disease, leaf blight, fruit rot, flower drop, and leaf spot [1,4]. Damping-off disease associated with *Sclerotium rolfsii* Sacc. (teleomorph: *Athelia rolfsii* (Curzi) C.C. Tu & Kimbr.) poses a serious threat to durian seedlings and may result in seedling mortality. Moreover, the transplantation of infected seedlings may facilitate the introduction and spread of the pathogen into plantation areas. The symptoms of the disease are similar to those caused by *Phytophthora palmivora* (Butler), but without the typical water-soaked lesions on the stem [5,6]. Instead, white mycelial growth is observed covering the stem and branches [7,8,9]. In severe cases, yellowing and defoliation may occur [10,11,12]. If left untreated, dense fungal growth, including the formation of brown mustard seed-like sclerotia, can be seen covering the infected stem and branches [13,14]. When cross-sections of infected tissues are examined, the vascular tissues (xylem) appear brown to dark brown. In addition, the root system is often severely affected, with fine roots deteriorating and detaching due to fungal infection [15,16].

Due to the severe damage caused by damping-off disease in durian seedlings (*Durio pinangianus*; specifically, ‘Durian Pa’ used as rootstock), farmers have become increasingly aware of the need to find effective control strategies. Chemical fungicides such as azoxystrobin, flutolanil, mefentrifluconazole, and tebuconazole are well-known active ingredients commonly used to prevent and control plant diseases [16,17,18]. However, the prolonged and repeated application of these chemical fungicides has contributed to the emergence of fungicide-resistant pathogens. As an alternative, *Trichoderma* spp. have been widely recognized as effective biocontrol agents due to their ability to suppress a broad spectrum of plant-pathogenic fungi through multiple mechanisms, including antibiotic production, competition, mycoparasitism, and the induction of plant resistance [5,10,12,19]. Trichoderma-based biological control offers an alternative approach to managing fungal pathogens [20,21].

The objective of this study was to evaluate the biocontrol potential of *Trichoderma* sp. THR3 against *Sclerotium rolfsii*, the causal agent of damping-off disease in durian (*Durio pinangianus*) seedlings, through in vitro and in vivo assessments. The aim was to explore a potentially effective and sustainable alternative to chemical fungicides for disease management in durian cultivation.

## 2. Results

### 2.1. Morphological and Molecular Characterization of Sclerotium rolfsii

**Figure 1:**
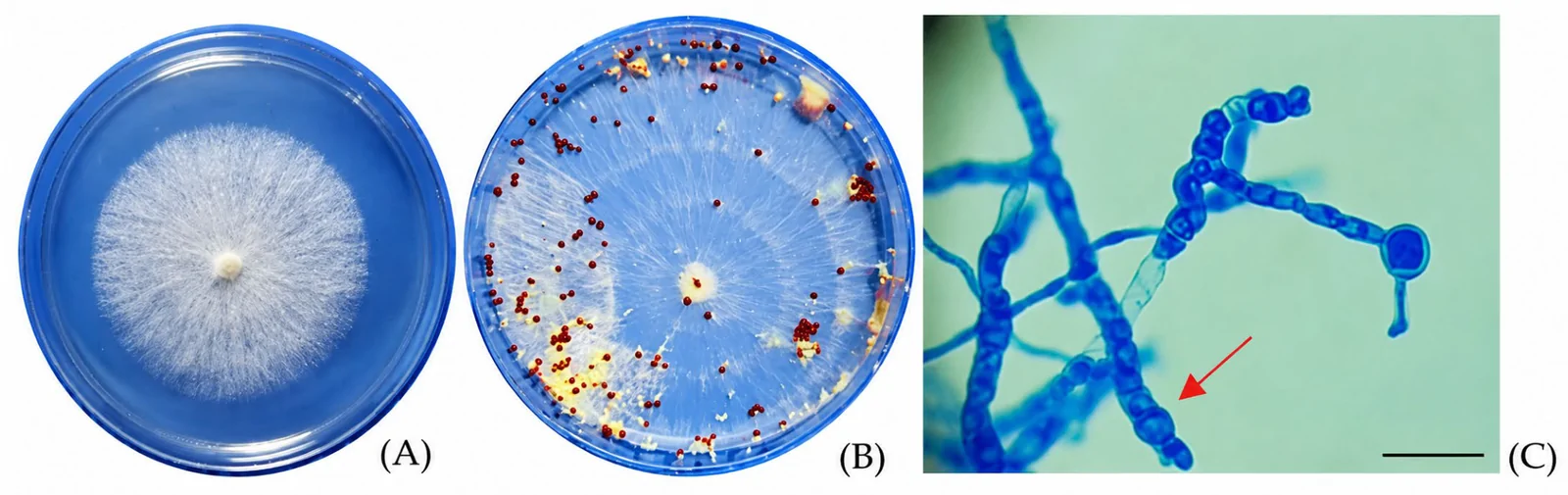
Morphological characteristics of *Sclerotium rolfsii* isolate SCO1: colony on potato dextrose agar (PDA) at 3 days (**A**), sclerotia (**B**), and mycelia (**C**).

**Figure 2:**
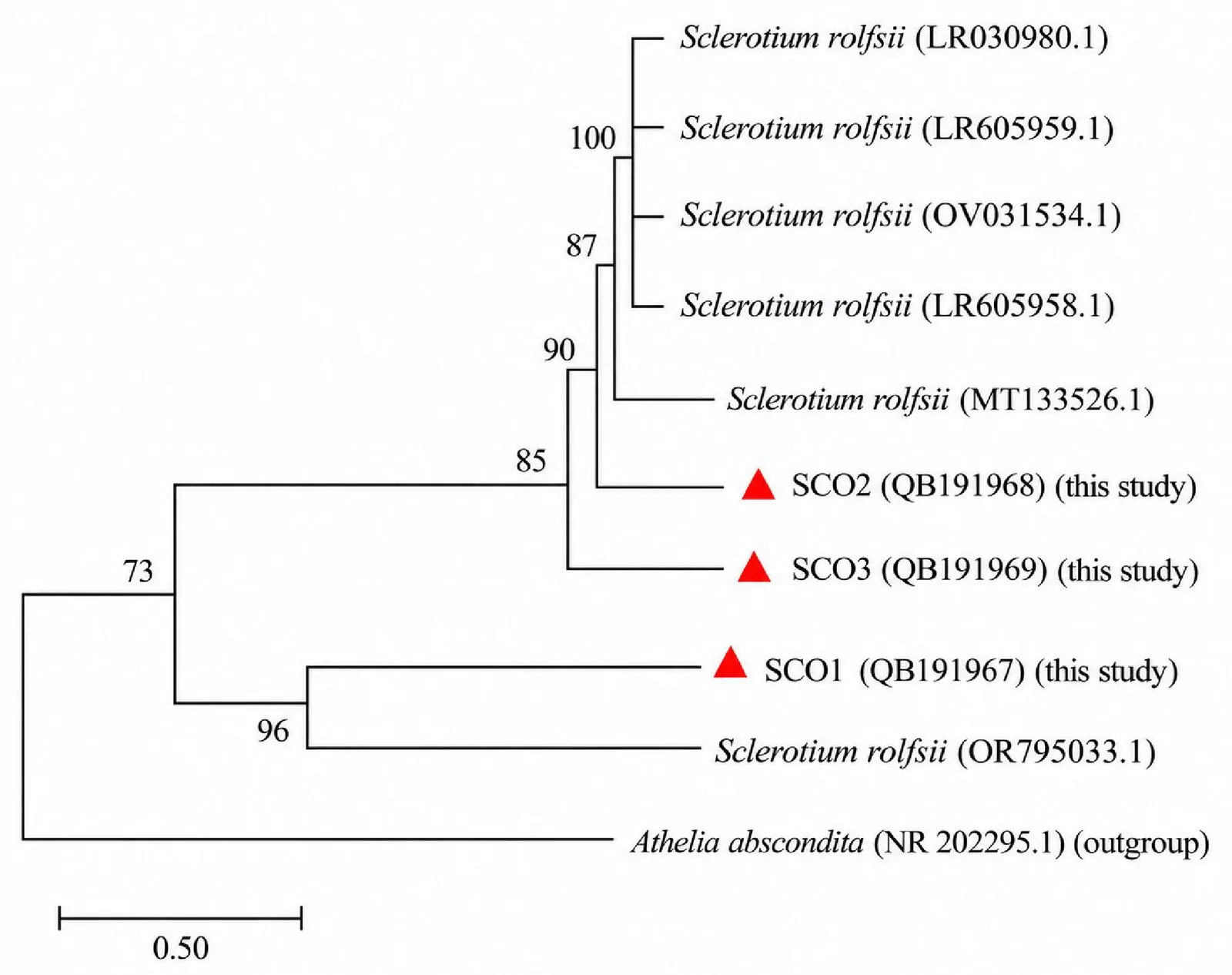
Maximum likelihood phylogenetic tree showing the relationships of *Sclerotium rolfsii* isolates SCO1 (QB191967), SCO2 (QB191968), and SCO3 (QB191969) with reference sequences retrieved from the NCBI GenBank database. The phylogenetic analysis was performed using the Tamura–Nei (TN93) nucleotide substitution model in MEGA version 11.0. Bootstrap support values based on 1000 replicates are indicated at the nodes. Red triangles indicate the isolates obtained in this study. The scale bar represents the estimated number of nucleotide substitutions per site.

Three isolates of *Sclerotium* spp.—SCO1, SCO2, and SCO3—were obtained from 50 samples of durian seedlings showing damping-off symptoms collected from orchards in Chanthaburi and Trat provinces. These isolates grew rapidly on potato dextrose agar (PDA), producing abundant white, cottony mycelia that later turned light brown to brown with age. The colonies typically radiated in a circular pattern. Small, round, white to brown sclerotia began to form after 3–5 days of incubation, usually near the edges of the colony or scattered across the surface. The sclerotia gradually matured and became hard, dark brown, and globose, measuring approximately 0.5–1.5 mm in diameter (Figure 1). The rDNA-ITS regions of isolates SCO1, SCO2, and SCO3 were amplified and sequenced using primers ITS5 (5′-GGAAGTAAAAGTCGTAACAAGG-3′) and ITS4 (5′-TCCTCCGCTTATTGATATGC-3′). Sequence comparison with reference sequences in GenBank showed that all three isolates exhibited high sequence similarity to *Sclerotium rolfsii*. Phylogenetic analysis based on the ITS sequences placed SCO1, SCO2, and SCO3 within the *S. rolfsii* group together with reference sequences retrieved from GenBank, while *Athelia abscondita* (NR_202295.1) formed a distinct outgroup (Figure 2). These molecular results, together with the morphological characteristics, supported the identification of SCO1, SCO2, and SCO3 as *S. rolfsii* (Figure 2).

### 2.2. Pathogenicity Test

**Figure 3:**
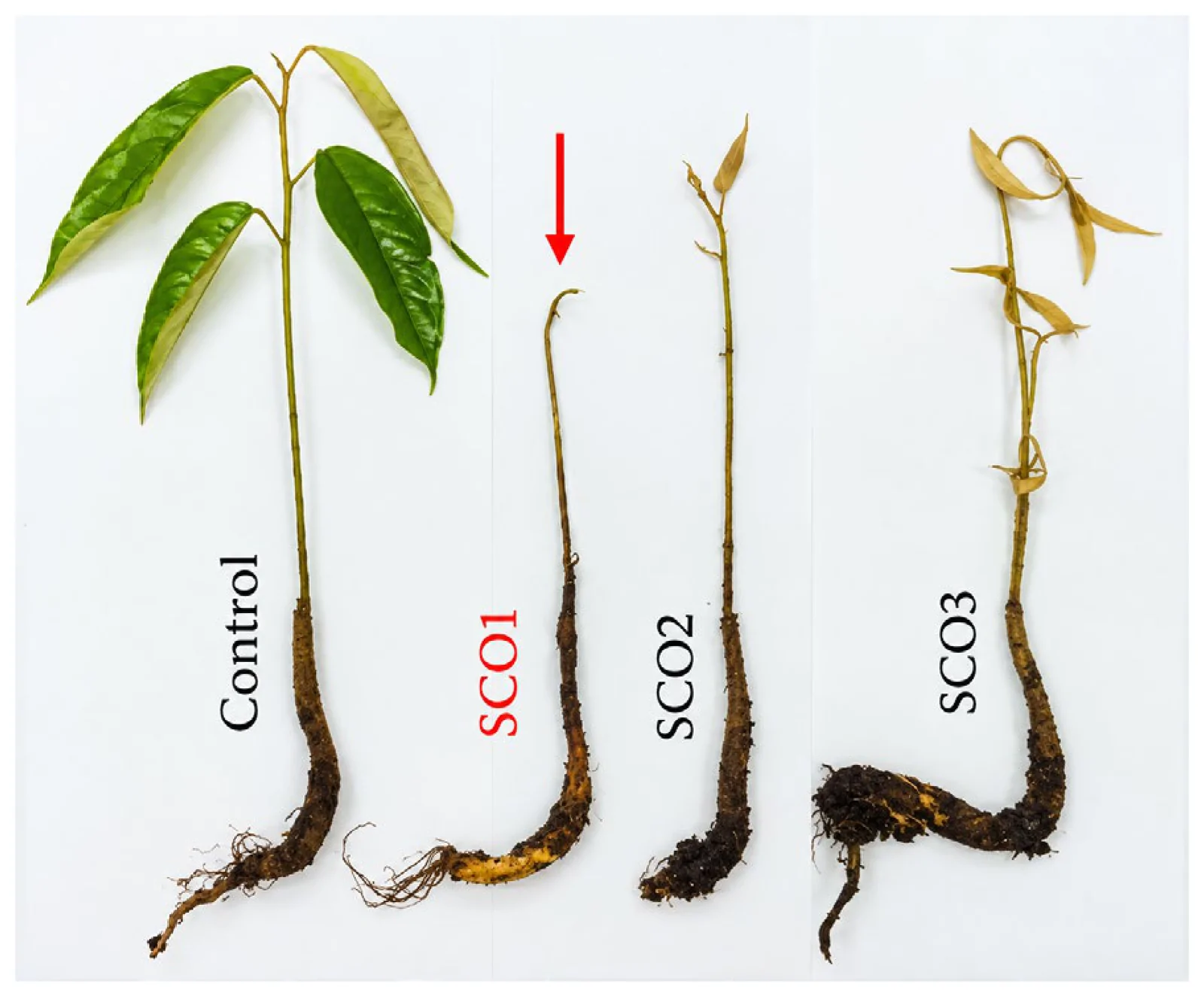
Pathogenicity test of *Sclerotium rolfsii* (SCO1, SCO2, and SCO3) on durian seedlings derived from seeds of ‘Durian Pa’ (*Durio pinangianus*) used as rootstock, with observations recorded 9 days after inoculation (DAI).

The pathogenicity of three *Sclerotium rolfsii* isolates (SCO1, SCO2, and SCO3) was evaluated on two-month-old durian seedlings over a 9-day assessment period (Table 1 and Figure 3). All three isolates caused disease symptoms, although disease incidence (DI) and disease severity (DS) varied among isolates and assessment times. SCO1 exhibited the greatest aggressiveness, with disease incidence reaching 100% as early as 3 days after inoculation (DAI) and remaining at this level throughout the experiment. Disease severity for SCO1 was 85.0% at 3 DAI, increased to 92.5% at 5 DAI, and reached 100% at 7 DAI, remaining unchanged at 9 DAI.

In contrast, SCO2 and SCO3 exhibited slower disease development. For SCO2, disease incidence was 60% at 3 and 5 DAI and increased to 80% at 7 DAI, remaining at this level through 9 DAI. Disease severity was 35.2% at 3 and 5 DAI, increased to 52.5% at 7 DAI, and reached 80.0% at 9 DAI. For SCO3, disease incidence increased from 40% at 3 DAI to 60% at 7 DAI and 80% at 9 DAI, whereas disease severity increased from 25.0% at 3 DAI to 47.5% at 5 and 7 DAI and 52.5% at 9 DAI. No disease symptoms were observed in the healthy control throughout the assessment period.

Overall, SCO1 caused the most rapid and severe disease development among the three isolates, supporting its selection for subsequent evaluation of biocontrol by *Trichoderma* sp. THR3.

### 2.3. Dual-Culture Test

The antagonistic activity of *Trichoderma* sp. THR3 against three *Sclerotium rolfsii* isolates (SCO1, SCO2, and SCO3) was evaluated using a dual-culture assay at 3, 5, and 7 days after inoculation (DAI) (Table 2). THR3 inhibited the mycelial growth of all three isolates, although the degree of inhibition varied among isolates and assessment times. At 3 DAI, the highest mycelial growth inhibition was observed against SCO1 (67.7%), followed by SCO2 (46.5%) and SCO3 (12.3%). Inhibition increased at 5 DAI to 75.4%, 65.4%, and 28.2%, respectively. At 7 DAI, the recorded inhibition values reached 88.7% for SCO1, 79.8% for SCO2, and 35.5% for SCO3.

The colony diameter measurements showed corresponding differences in fungal growth. At 3 DAI, control colony diameters were 2.8, 3.0, and 6.0 cm for SCO1, SCO2, and SCO3, respectively, whereas the corresponding diameters in dual culture with THR3 were 0.90, 1.60, and 5.26 cm. At 5 DAI, the control colony diameters increased to 4.0, 5.0, and 8.0 cm, while the corresponding dual-culture diameters were 0.98, 1.73, and 5.74 cm. By 7 DAI, all control colonies had reached the 9-cm Petri dish boundary, whereas the measured colony diameters in dual culture were 1.01, 1.81, and 5.80 cm for SCO1, SCO2, and SCO3, respectively.

**Figure 4:**
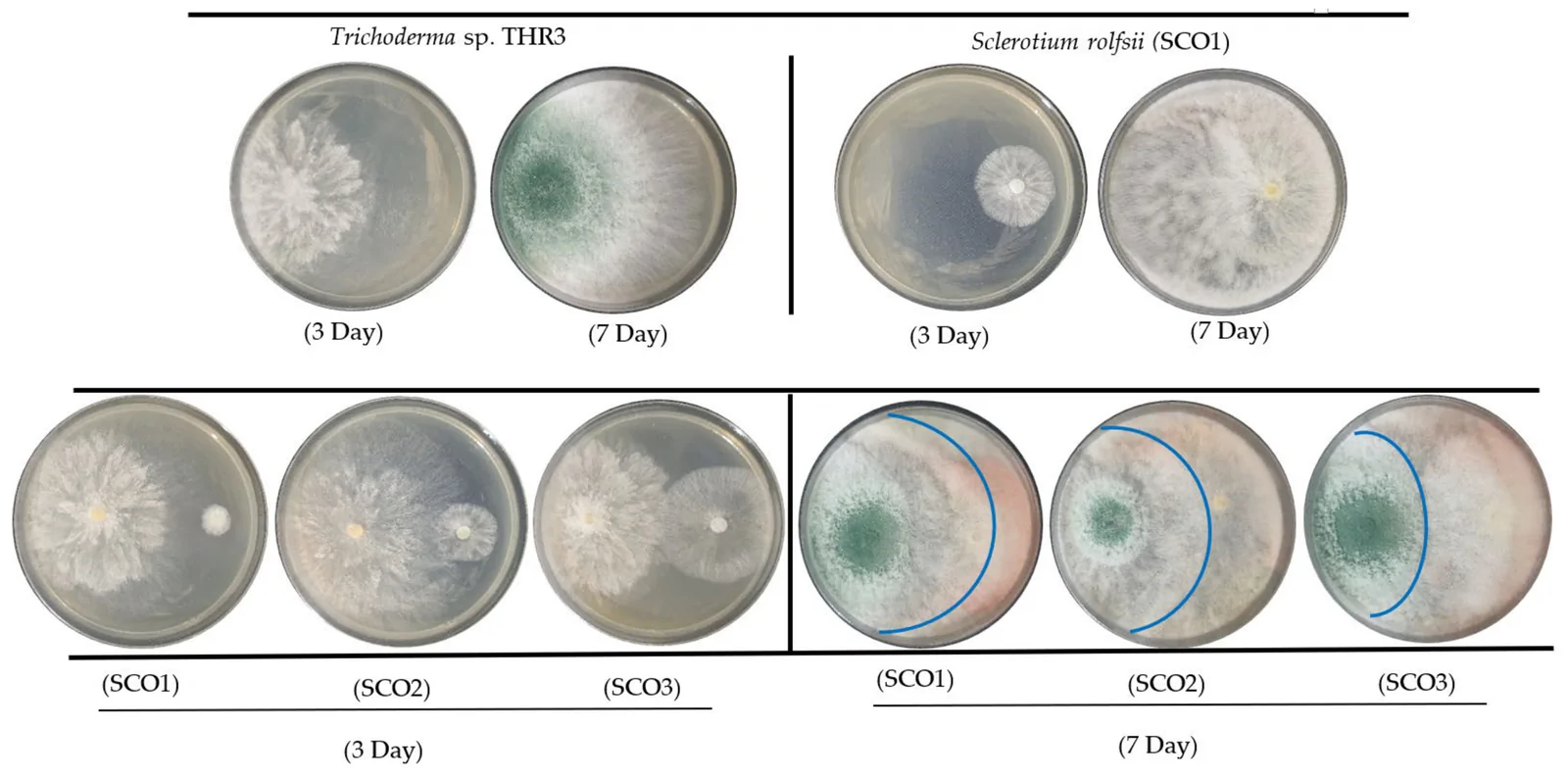
Colony growth and interactions between *Trichoderma* sp. THR3 and *Sclerotium rolfsii* isolates in monoculture and dual-culture assays. The upper panels show monocultures of THR3 and *S. rolfsii* SCO1 at 3 and 7 days after inoculation (DAI). The lower panels show dual-culture interactions between THR3 and three *S. rolfsii* isolates (SCO1, SCO2, and SCO3) at 3 and 7 DAI. The blue curved lines indicate the visually identified boundaries of THR3 overgrowth onto *S. rolfsii* colonies at 7 DAI.

Overall, THR3 showed the greatest inhibitory activity against SCO1, followed by SCO2 and SCO3. However, because all control colonies reached the plate boundary by 7 DAI, the inhibition percentages at this assessment point should be interpreted cautiously, as further control colony expansion was physically restricted by the Petri dish diameter (Figure 4).

### 2.4. Biocontrol of Damping-Off Disease in Durian Seedlings

The healthy control (Tr1) remained symptomless throughout the experimental period. Plants treated with *Trichoderma* sp. THR3 alone (Tr2) exhibited a constant disease incidence (DI) of 20%, whereas disease severity (DS) increased from 10% at 3 DAI to 20% at 5 DAI and remained unchanged thereafter (Table 3). Plants inoculated with *Sclerotium rolfsii* SCO1 alone (Tr3) exhibited progressively increasing disease incidence and severity, reaching 100% for both parameters at 9 DAI. In contrast, plants treated with *Trichoderma* sp. THR3 and inoculated with SCO1 (Tr4) exhibited lower numerical values of DI and DS throughout the observation period. At 9 DAI, disease incidence (DI) and disease severity (DS) reached 100% in the pathogen-inoculated control (Tr3), whereas the corresponding values in plants treated with *Trichoderma* sp. THR3 (Tr4) were 60% and 50%, respectively. This represented a 50% numerical reduction in final disease severity relative to Tr3. However, the difference in DI between Tr3 and Tr4 was not statistically significant (*p* > 0.05), and the reduction in DS should be interpreted cautiously because the direct pairwise comparison has not been conclusively established.

Longitudinal permutation analysis indicated a significant overall effect of treatment on disease incidence (DI; *p* = 0.0147), whereas the effects of assessment time (*p* = 0.1248) and the treatment × time interaction (*p* = 0.5603) were not statistically significant. In contrast, treatment significantly affected disease severity (DS) (*p* = 0.0446), as did assessment time (*p* < 0.001) and their interaction (*p* = 0.0008) (Table 4). These results indicate that disease severity varied among treatments and that its progression over time differed across treatments. However, the significant overall treatment effects do not necessarily indicate significant differences between the pathogen-inoculated control (Tr3) and the THR3-treated group (Tr4).

**Figure 5:**
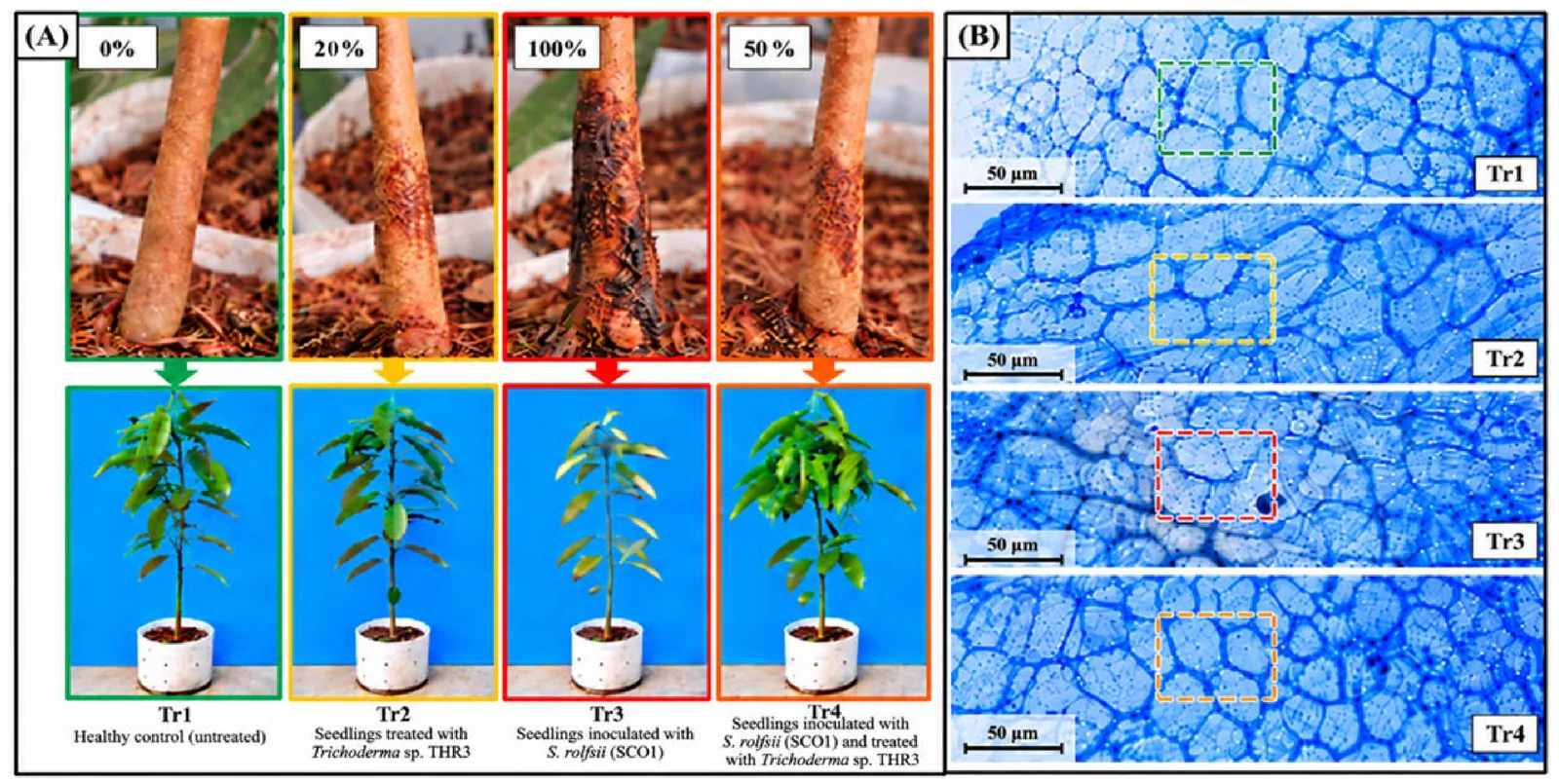
Macroscopic symptoms and microscopic characteristics of stem tissues of one-year-old grafted durian plants under different treatments at 9 days after inoculation (DAI). (**A**) Representative stem-base symptoms and whole-plant responses; percentages denote the reported treatment-level disease severity in Table 3: Tr1, healthy control (untreated; 0% disease severity); Tr2, seedlings treated with *Trichoderma* sp. THR3 (20%); Tr3, seedlings inoculated with *Sclerotium rolfsii* SCO1 (100%); and Tr4, seedlings inoculated with *S. rolfsii* SCO1 and treated with *Trichoderma* sp. THR3 (50%). Arrows indicate the inoculation sites. (**B**) Representative microscopic observations of stem tissues from the corresponding treatments (Tr1–Tr4). Tissue organization was largely preserved in Tr1 and Tr2, whereas Tr3 exhibited more pronounced alterations in tissue organization. In Tr4, tissue alterations appeared less pronounced than those observed in Tr3. Scale bars = 50 μm.

To further investigate the mild symptoms observed in plants treated with *Trichoderma* sp. THR3 alone (Tr2), we conducted an additional experiment using a wounded mock-inoculated control (S-Tr1) and wounded plants treated with THR3 (S-Tr2), without inoculation with *Sclerotium rolfsii* SCO1. Symptoms were observed in both treatments. At 9 days after treatment, S-Tr1 exhibited disease incidence (DI) and disease severity (DS) values of 60% and 30%, respectively, whereas S-Tr2 exhibited corresponding values of 20% and 15%. The disease assessment results are summarized in Table S1, and the associated fungal observations are presented in the Supplementary Materials. These findings indicate that stem-base symptoms could develop following wounding even without deliberate inoculation with either THR3 or SCO1. However, the causal agent responsible for these symptoms was not established, and the pathogenic potential of THR3 could not be conclusively determined from these observations. Microscopic examination of durian stem tissues revealed differences in tissue organization among the four treatments (Figure 5B). Tr1 exhibited largely intact and well-organized cellular structures, with clearly defined cell boundaries. Similarly, Tr2 showed relatively preserved tissue organization, despite the mild disease symptoms observed macroscopically (DS = 20%). In contrast, Tr3 exhibited more pronounced irregularities in cellular arrangement and tissue organization, corresponding to severe stem-base necrosis and the highest disease severity (DS = 100%). In Tr4, cellular structures appeared comparatively better preserved, with less pronounced tissue alterations than those observed in Tr3, consistent with the lower disease severity (DS = 50%) recorded following treatment with *Trichoderma* sp. THR3.

## 3. Discussion

This study evaluated the efficacy of *Trichoderma* sp. THR3 in controlling *Sclerotium rolfsii* associated with damping-off disease in durian seedlings. The investigation began by isolating the fungal pathogen from infected durian seedling tissues exhibiting typical damping-off symptoms. Three isolates were obtained and initially identified as *Sclerotium* spp. and designated SCO1, SCO2, and SCO3. Subsequent molecular characterization confirmed that all three isolates were *Sclerotium rolfsii*, with 100% sequence similarity to reference sequences. These results are consistent with previous studies by Pacheco et al. [22], Nonthapa et al. [23], Sutthisa et al. [19], and Naznin and Monjil [24], which reported *S. rolfsii* as a common and aggressive soil-borne pathogen in tropical and subtropical regions. In addition to durian seedlings, damping-off disease caused by *S. rolfsii* has been reported in a broad range of crops, including legumes such as soybean and lentil [14,24], onion [25], sugar beet [7], chili [8,15], cucumber [26], peanuts [11,27], and various vegetables [23]. Moreover, the pathogen has been found not only in food crops but also in ornamental plants such as carnations [28] and sunflower [16], highlighting its broad host range and agricultural importance.

The three *Sclerotium rolfsii* isolates (SCO1, SCO2, and SCO3) were subjected to pathogenicity testing on two-month-old durian seedlings to evaluate their ability to cause damping-off disease. The results revealed that all three isolates were pathogenic to durian seedlings. Among the three isolates, SCO1 exhibited the highest aggressiveness, with disease incidence reaching 100% at 3 days after inoculation (DAI) and remaining unchanged throughout the experiment. Disease severity increased from 85% at 3 DAI to 92.5% at 5 DAI and reached 100% at 7 DAI, remaining at this level until 9 DAI. SCO2 and SCO3 caused comparatively lower levels of disease incidence and severity, ranking second and third, respectively. These findings are consistent with the study by Billah et al. [29], who reported that *Sclerotium rolfsii* is pathogenic to a wide range of plant species, including sunflower, moonbeam, betel vine, lentil, sugar beet, tomato, sweet pumpkin, maize, chickpea, potato, soybean, oat, and several ornamental plants. Pathogens associated with sclerotial diseases cause various symptoms, such as damping-off of seedlings, stem canker, crown blight, and rots of roots, crowns, bulbs, tubers, and fruits. Furthermore, the mycelia of *S. rolfsii* spread rapidly and can persist in soil for extended periods in the form of sclerotia.

Isolate THR3 was initially identified as *Trichoderma asperellum* based on ITS sequence analysis, which revealed 100% sequence identity (602/602 bp) with a GenBank sequence annotated as *T. asperellum* (accession number KM456214.1). The nucleotide sequence of THR3 was deposited in GenBank under accession number QB184922. However, ITS sequence analysis alone may not provide sufficient resolution for definitive species-level identification within the genus *Trichoderma*. Therefore, the isolate was conservatively designated as *Trichoderma* sp. THR3 in the present study. Further molecular characterization using additional markers, particularly tef1α and rpb2, would provide stronger support for species-level identification. Although the species-level identity of THR3 remains unresolved, this taxonomic uncertainty does not invalidate the biological control activity observed for isolate THR3 against *S. rolfsii* under the experimental conditions.

The antagonistic activity of *Trichoderma* sp. THR3 against the three *Sclerotium rolfsii* isolates (SCO1, SCO2, and SCO3) associated with damping-off in durian seedlings was evaluated using a dual-culture assay. The results demonstrated that *Trichoderma* sp. THR3 inhibited the mycelial growth of all three *S. rolfsii* isolates, with the greatest inhibition observed for SCO1 (88.7%), followed by SCO2 and SCO3. The antagonistic activity observed in the dual-culture assay may involve multiple biological interactions. Mycelial growth inhibition was observed at 3 days after inoculation (DAI), followed by overgrowth of the pathogen colonies by THR3 between 5 and 7 DAI. These observations represent phenotypic interaction patterns rather than direct evidence of specific antagonistic mechanisms. Previous studies have demonstrated that *Trichoderma* species can produce diverse specialized metabolites potentially involved in antagonism, microbial interactions, and ecological adaptation [30,31,32]. However, the production and biological functions of these compounds are strain-dependent and require experimental verification. In the present study, no chemical characterization, metabolite profiling, or functional assays were performed to identify antifungal compounds produced by THR3. Therefore, the involvement of antibiosis, competition, or other antagonistic mechanisms remains hypothetical. Further studies involving metabolomic analyses, assays of volatile and non-volatile metabolites, and functional approaches are required to clarify the mechanisms underlying the observed inhibition of *S. rolfsii*. These results agree with those of Pandey and Devkota [33], who reported high inhibition rates of *S. rolfsii* by *Trichoderma* spp. in dual-culture assays, reaching 90.50%, 94.67%, 97.75%, and 100% at 2, 3, 4, and 5 days after inoculation, respectively. Similarly, Liu et al. [5] demonstrated that *Trichoderma semiorbis* strain FJ059 completely inhibited the mycelial growth of *S. rolfsii* and was able to kill 80% of sclerotia on PDA plates after 10 days of incubation. Additional evidence was provided by Rawat and Tewari [34], who observed that *Trichoderma* caused significant structural alterations in *S. rolfsii* cells, including cytoplasmic retraction, aggregation, disintegration, and deformation of the cell wall, ultimately resulting in the collapse of sclerotial integrity. Rivera-Mendez et al. [25] also reported strong antagonistic activity of *T. asperellum* strains against *S. cepivorum*, with the BCC1 strain achieving 81% and 90% inhibition in dual-culture and cellophane membrane assays, respectively. Several other *Trichoderma* species have also shown antagonistic potential against *S. rolfsii*, including *T. longibrachiatum* [6,35], *T. reesei* [36,37], and *T. harzianum* [38,39], further supporting the broad biocontrol potential of this genus. Several studies have also investigated bacterial antagonists, particularly *Bacillus* species, for controlling *Sclerotium rolfsii*. Strains such as *B. subtilis*, *B. siamensis*, *B. amyloliquefaciens*, *B. velezensis*, and *B. atrophaeus* have been shown to inhibit the growth of *S. rolfsii*, with inhibition rates reaching 58–93% [9,12,18,40]. Chemical control approaches have also been evaluated. Fungicides such as azoxystrobin, flutolanil, mefentrifluconazole, and tebuconazole have been tested for their efficacy against *S. rolfsii*. Among them, mefentrifluconazole was reported to provide favorable control efficacy by significantly reducing both the infection rate and the population density of *S. rolfsii* [10,41].

Interestingly, mild symptoms were observed in seedlings treated with *Trichoderma* sp. THR3 alone (Tr2), whereas the untreated healthy control (Tr1) remained symptomless. The cause of these symptoms was not established, and the observations alone neither demonstrate nor exclude the pathogenicity of THR3. Microscopic observations showed that tissue organization in Tr2 was largely preserved and comparable to that of the healthy control (Tr1), whereas more pronounced tissue alterations were observed in seedlings inoculated with *Sclerotium rolfsii* SCO1 (Tr3) (Figure 5). However, Tr2 seedlings received five needle wounds in the stem-base region, whereas Tr1 remained unwounded. Furthermore, the soil was heat-treated at 100 °C for 1 h without experimental verification of complete sterility, and the seedlings were maintained under nursery conditions. Therefore, mechanical injury and potential opportunistic microbial infection cannot be excluded as possible contributors to the symptoms observed in Tr2. Because a wounded mock-inoculated control was not included, the present experiment could not distinguish these possible causes from effects associated with THR3 application. This limitation highlights the need for further safety evaluation of THR3 using appropriate wounded controls and re-isolation of microorganisms from symptomatic tissues. Although THR3 exhibited strong antagonistic activity against SCO1 in the dual-culture assay, its disease-suppressive effect in durian seedlings was more limited. THR3 inhibited the mycelial growth of SCO1 by 88.7% under in vitro conditions, whereas the combined treatment (Tr4) resulted in a 50% numerical reduction in final disease severity relative to the pathogen-inoculated control (Tr3). However, these values represent different response variables and should not be interpreted as directly comparable measures of efficacy. The differences between in vitro and in planta responses may reflect the complexity of interactions among the pathogen, antagonist, host plant, and surrounding environment. In particular, pathogen establishment, host physiological responses, environmental conditions, and the ability of THR3 to colonize and persist at the infection site may influence disease suppression. Furthermore, THR3 was applied three days after SCO1 inoculation, which may have affected its effectiveness. Nevertheless, these factors were not directly investigated in the present study and should be regarded as possible explanations rather than confirmed mechanisms. Previous studies have demonstrated the potential of *Trichoderma* species to suppress diseases caused by *S. rolfsii* and related pathogens in different host plants [24,42,43,44]. However, differences in host species, pathogen isolates, experimental conditions, and assessment methods limit direct comparisons of biological control efficacy across studies. Similarly, plant growth-promoting rhizobacteria, including *Bacillus megaterium* and *Pseudomonas fluorescens*, have shown potential for suppressing southern blight and promoting plant growth in chili [40]. These findings highlight the broader potential of microbial antagonists for disease management, although their effectiveness must be validated under the specific crop and production conditions in which they are intended to be used.

Several limitations of the present study should be acknowledged. Although three *Sclerotium rolfsii* isolates (SCO1, SCO2, and SCO3) were evaluated in the pathogenicity and dual-culture assays, the in planta biocontrol experiment involved only one pathogen isolate (SCO1) and one biocontrol isolate (*Trichoderma* sp. THR3). Furthermore, the experiment did not include a chemical fungicide positive control or a reference biocontrol strain, preventing direct comparisons of THR3 with conventional fungicide treatments or other biological control agents. The biocontrol evaluation was conducted as a single pot experiment without independent experimental repetition, which limits the generalizability of the findings across different experimental conditions. In addition, the pathogenicity assay used two-month-old durian seedlings, whereas the biocontrol assay was conducted using one-year-old grafted durian plants. Therefore, the observed efficacy of THR3 should be interpreted specifically within the experimental conditions and plant developmental stage evaluated in this study.

Further studies are needed to evaluate the practical applicability and safety of THR3 under durian nursery conditions. Future experiments should include wounded mock-inoculated controls, additional *S. rolfsii* isolates, reference biocontrol strains, and chemical fungicide controls, together with independently repeated trials using younger durian seedlings representing the growth stages most susceptible to damping-off. Evaluation of different application timings, THR3 establishment and persistence, and potential effects on plant health would provide stronger evidence for its suitability as a biological control agent.

## 4. Materials and Methods

### 4.1. Trichoderma sp. THR3

*Trichoderma* sp. THR3 was obtained from the Plant Pathology Laboratory, Department of Plant Production and Landscape, Faculty of Agro-Industrial Technology, Rajamangala University of Technology Tawan-Ok, Chanthaburi, Thailand. The isolate was originally collected from rhizosphere soil of volcanic origin in a durian field in Chanthaburi Province, Thailand. It was preserved in 20% glycerol (99.5% purity; KemAus, Australia) at −20 °C in 2023 and subsequently recovered for experimental evaluation in 2025. Following storage, the isolate was revived on potato dextrose agar (PDA; Cat. No. M096-500G; HiMedia Laboratories Pvt. Ltd., Thane, India), and its viability was confirmed by successful mycelial growth. The identity of the recovered isolate was assessed by examining its colony morphology and microscopic characteristics for consistency with those of the original THR3 culture before its use in subsequent experiments.

The isolate was initially identified as *Trichoderma asperellum* based on ITS sequence analysis. BLAST (National Center for Biotechnology Information, Bethesda, MD, USA; https://blast.ncbi.nlm.nih.gov/Blast.cgi; accessed on 16 October 2026) analysis revealed 100% sequence identity (602/602 bp) with a GenBank sequence annotated as *T. asperellum* (accession number KM456214.1). The supporting ITS sequence identification report obtained from Macrogen is provided in Supplementary Figure S2. The nucleotide sequence of isolate THR3 was deposited in GenBank under accession number QB184922. Given the limited resolution of ITS sequences for definitive species-level identification within the genus *Trichoderma*, the isolate was conservatively designated as *Trichoderma* sp. THR3 in the present study.

### 4.2. Isolation and Characterization of Sclerotium rolfsii

**Figure 6:**
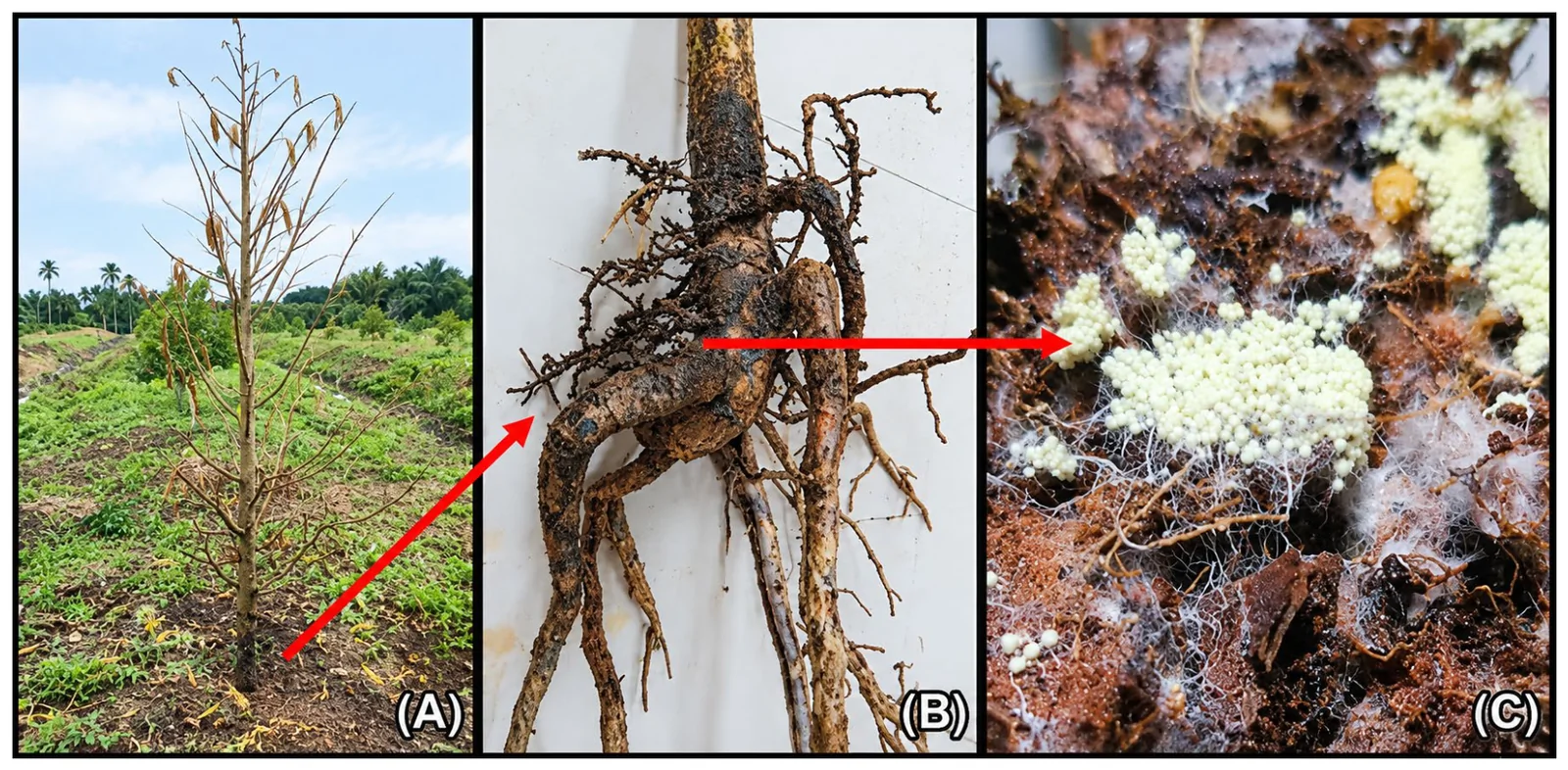
Damping-off symptoms in durian seedlings. (**A**–**C**) Affected seedlings show wilting, leaf yellowing, and collapse at the stem base, which becomes dark and water-soaked and rapidly decays, leading to plant death. (**C**) Mycelia and sclerotia on infected tissues.

Grafted durian plants showing damping-off symptoms were collected from Khao Wua Phloi Waen Subdistrict, Tha Mai District, Chanthaburi Province, and Bo Phloi Subdistrict, Bo Rai District, Trat Province (Figure 6). These plants consisted of ‘Durian Pa’ (*Durio pinangianus*) seedling rootstocks grafted with ‘Monthong’ (*Durio zibethinus*) scions. Fungal isolation was performed using the tissue transplanting method. Infected root segments were aseptically excised, trimmed into 1–1.5 cm sections, and surface-disinfested in a 10% (*v*/*v*) commercial bleach solution (Clorox; 8.25% sodium hypochlorite stock solution; The Clorox Company, Oakland, CA, USA) for 1 min. After being rinsed three times with sterile distilled water, the segments were plated on potato dextrose agar (PDA). Morphological identification of *Sclerotium rolfsii* was based on the criteria described by Sanabria-Velazquez et al. [6], focusing on the structure of mycelia and sclerotia. Pathogenicity was assessed on durian seedlings using the method described by Billah et al. [29].

### 4.3. Molecular Characterization

Molecular identification of isolates SCO1, SCO2, and SCO3 was performed following DNA extraction using the PrepMan™Ultra Sample Preparation Reagent (Applied Biosystems, Thermo Fisher Scientific, Waltham, MA, USA). The extracted DNA samples were sent to Macrogen Inc. (Seoul, Republic of Korea) for sequencing. The internal transcribed spacer (ITS) region of nuclear ribosomal DNA (rDNA), encompassing ITS1, the 5.8S rRNA gene, and ITS2, was amplified using the universal fungal primers ITS5 (5′-GGAAGTAAAAGTCGTAACAAGG-3′) and ITS4 (5′-TCCTCCGCTTATTGATATGC-3′), as described by White et al. [45]. The resulting sequences were analyzed using the BLAST algorithm against the NCBI GenBank database for species-level identification. The nucleotide sequences deposited in GenBank comprised the complete ITS1–5.8S–ITS2 region and a partial sequence of the large subunit ribosomal RNA (LSU) gene. The corresponding accession numbers are QB191967, QB191968, and QB191969 for *S. rolfsii* isolates SCO1, SCO2, and SCO3, respectively. Phylogenetic analysis was conducted using the maximum likelihood (ML) method under the Tamura–Nei (TN93) nucleotide substitution model in Molecular Evolutionary Genetics Analysis (MEGA) version 11.0. Bootstrap analysis with 1000 replicates was performed to assess branch support. *Athelia abscondita* (NR_202295.1) was selected as the outgroup for the phylogenetic analysis of *S. rolfsii* isolates.

### 4.4. Pathogenicity Test

Three *Sclerotium rolfsii* isolates (SCO1, SCO2, and SCO3), obtained and identified as described above, were used in the pathogenicity test. Each isolate was cultured on potato dextrose agar (PDA) for 7 days. For pathogen inoculation, all PDA medium from one plate colonized by the respective isolate was thoroughly mixed into the soil around the base of each two-month-old durian seedling to ensure contact between the pathogen and the root zone. For the control treatment, an equivalent amount of sterile, uninoculated PDA medium (one plate per pot) was mixed into the soil using the same procedure. The experiment was arranged in a completely randomized design (CRD) comprising four treatments (SCO1, SCO2, SCO3, and an uninoculated control), with ten replications per treatment and one seedling per replication. Each seedling was enclosed in a plastic bag to maintain conditions favorable for disease development. Disease incidence (DI) was calculated as the percentage of seedlings exhibiting disease symptoms relative to the total number of seedlings assessed. Disease severity (DS) was evaluated using a five-level rating scale (0–4), adapted from Billah et al. [29], as follows: Level 0, no disease symptoms at the stem base; Level 1, small necrotic spots at the stem base, with approximately 25% of leaves showing yellowing; Level 2, browning of the stem base, with 26–50% of leaves yellowing; Level 3, dark brown stem base with wilting symptoms and 51–75% leaf drop; and Level 4, blackening and rotting of the stem base, accompanied by 76–100% defoliation.

Disease incidence and disease severity were calculated using the following equations:

DI (%) = (Number of symptomatic seedlings/Total number of seedlings assessed) × 100

DS (%) = [Σ(n_i_ × v_i_)/(N × 4)] × 100

where n_i_ represents the number of seedlings assigned to each disease severity category, v_i_ represents the corresponding severity score, and N represents the total number of seedlings assessed.

The experiments were conducted in an open-sided nursery greenhouse covered with a transparent plastic roof to protect the seedlings from rainfall. The greenhouse was exposed to natural daylight and ambient environmental conditions. The temperature inside the greenhouse was approximately 28–35 °C, while relative humidity generally ranged from 60% to 85%, depending on weather conditions and the time of day. These values represent approximate environmental conditions rather than continuously monitored measurements.

Following symptom development, fungal pathogens were re-isolated from symptomatic tissues of durian seedlings inoculated with each of the three *Sclerotium rolfsii* isolates (SCO1, SCO2, and SCO3). The recovered fungi were cultured on potato dextrose agar (PDA), and their colony morphology and microscopic hyphal characteristics were examined and compared with those of the corresponding original isolates used for inoculation.

### 4.5. Dual-Culture Test

The antagonistic effectiveness of *Trichoderma* sp. THR3 against *Sclerotium rolfsii* isolates SCO1, SCO2, and SCO3 was evaluated using the dual-culture technique on potato dextrose agar (PDA) medium, following the method of Dennis and Webster [46]. To initiate the test, 20 mL of PDA was poured into sterile Petri dishes and allowed to solidify. Five-day-old cultures of both *Trichoderma* sp. THR3 and *S. rolfsii* were used. Mycelial discs (6 mm in diameter), taken from the actively growing edge of each fungal culture, were placed opposite each other at equal distances from the edge of the Petri plate. For the control treatment, a single 6-mm mycelial disc of each *S. rolfsii* isolate was placed at the same position as the corresponding pathogen disc in the dual-culture treatment, without inoculation with *Trichoderma* sp. THR3. Control and dual-culture plates were incubated under identical conditions and evaluated at the same assessment times.

Each treatment, along with the control (inoculated with *S. rolfsii* alone), consisted of five replicates. The experiment was carried out under aseptic conditions, and the plates were incubated at 25 ± 1 °C for 10 days. The radial growth of *S. rolfsii* was measured twice daily, and mean values were calculated. The percentage of mycelial growth inhibition was determined by comparing the average radial growth in the treated plates with that in the control, using the formula: I = (C − T)/C × 100, where I is the inhibition percentage, C is the average radial growth of *S. rolfsii* in the control plates, and T is the average radial growth in the presence of *Trichoderma* sp. THR3 [5].

### 4.6. Biocontrol of Damping-Off Disease in Durian Seedlings

**Figure 7:**
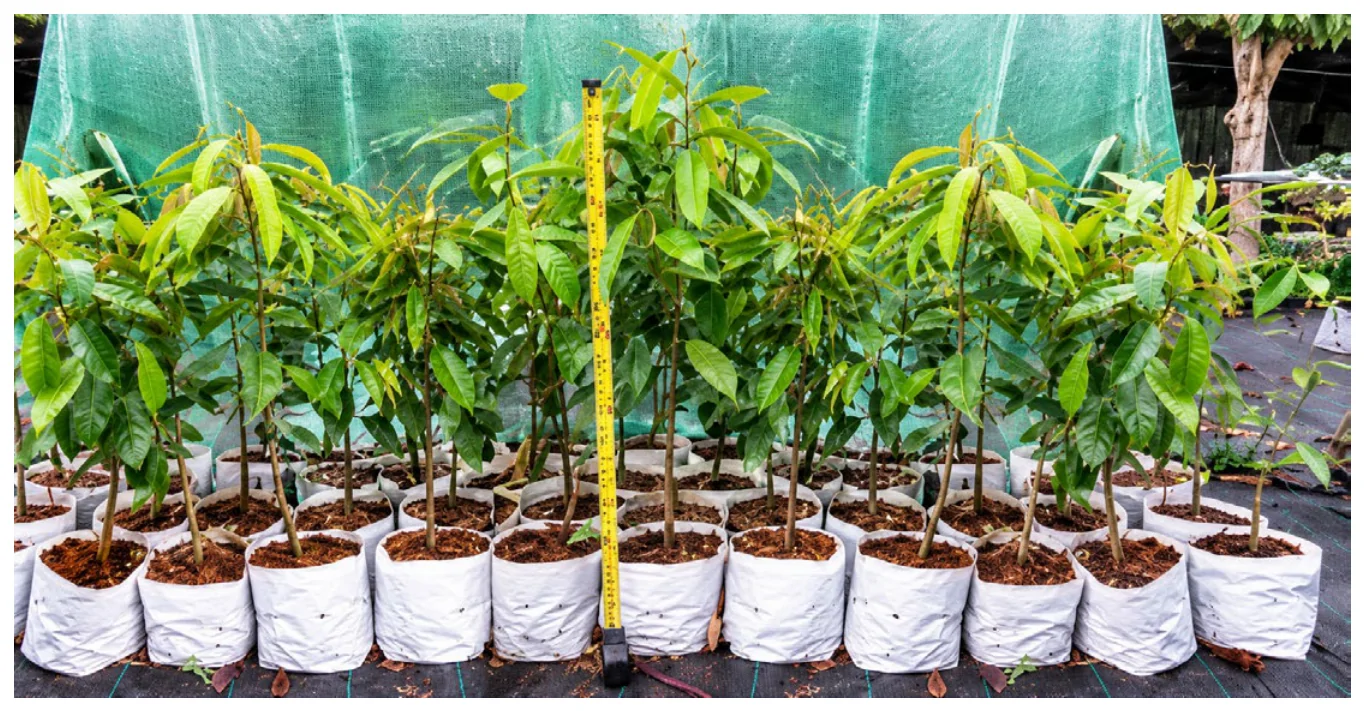
One-year-old grafted durian plants used in the biocontrol experiment.

The efficacy of *Trichoderma* sp. THR3 in controlling damping-off disease of durian seedlings caused by *Sclerotium rolfsii* was evaluated. One-year-old grafted durian seedlings, derived from seeds of ‘Durian Pa’ (*Durio pinangianus*) used as rootstock and grafted with the Monthong cultivar (*Durio zibethinus*) as the scion, were transplanted into plastic pots containing 2.5 kg of heat-treated soil. The soil was heat-treated in a hot-air oven (UF110; Memmert GmbH + Co. KG, Schwabach, Germany) at 100 °C for 1 h before transplanting. Complete soil sterility was not experimentally verified. The experiment was arranged in a completely randomized design (CRD) with four treatments and five replicates per treatment, using one seedling per replicate, for a total of 20 seedlings (Figure 7). *Sclerotium rolfsii* SCO1 was cultured on potato dextrose agar (PDA) for 7 days before inoculation. Mycelial discs (0.5 cm in diameter) were excised from the cultures using a sterile cork borer and placed directly on the stem-base region of the ‘Durian Pa’ rootstock, below the graft union. To facilitate infection, five wounds were made around the stem-base region of the rootstock using a sterile needle. The inoculation sites were then sealed with Parafilm M laboratory film (Amcor Flexibles North America, Inc., Neenah, WI, USA) to maintain close contact between the mycelial discs and the wounded rootstock tissue.

*Trichoderma* sp. THR3 was cultured on potato dextrose agar (PDA) for 7 days. Conidia were harvested from the cultures using sterile distilled water to prepare a conidial suspension. The conidial concentration was determined using an improved Neubauer bright-line hemocytometer (model BOE 14; Boeckel + Co. (GmbH + Co.) KG, Hamburg, Germany) and adjusted to 1 × 10^7^ conidia/mL. The prepared suspension was applied by spraying 10 mL per seedling, ensuring coverage of the entire plant. The experiment consisted of four treatments: Tr1, healthy control (untreated and unwounded); Tr2, seedlings treated with *Trichoderma* sp. THR3 without pathogen inoculation; Tr3, seedlings inoculated with *S. rolfsii* SCO1; and Tr4, seedlings inoculated with *S. rolfsii* SCO1 and subsequently treated with *Trichoderma* sp. THR3. Five needle wounds were made at the stem-base region of seedlings in Tr2, Tr3, and Tr4, whereas Tr1 remained unwounded. For Tr4, a conidial suspension of *Trichoderma* sp. THR3 (1 × 10^7^ conidia mL^−1^; 10 mL per seedling) was applied to the stem-base region of the ‘Durian Pa’ rootstock three days (72 h) after inoculation with *S. rolfsii* SCO1. The THR3-only treatment (Tr2) received the same conidial suspension without pathogen inoculation. Thus, the combined treatment was designed to evaluate the post-inoculation biocontrol potential of THR3 against *S. rolfsii* SCO1. The biocontrol experiment was conducted under open-sided nursery greenhouse conditions, with natural daylight, approximate temperatures of 28–35 °C, and relative humidity of 60–85%, consistent with the experimental conditions described in Section 4.4.

At 9 days after inoculation (DAI), stem tissue samples were collected from the stem-base regions of durian seedlings to examine anatomical alterations associated with disease development. Thin transverse sections were manually prepared using a sterile, sharp razor blade and transferred onto clean glass microscope slides. The sections were stained with lactophenol cotton blue (LPCB; HiMedia Laboratories Pvt. Ltd., Thane, India)) to facilitate the visualization of fungal structures and tissue organization. Coverslips were gently placed over the specimens, and the prepared slides were briefly warmed to facilitate the removal of trapped air bubbles and improve penetration of the staining solution into the tissue sections. After cooling to room temperature, the slides were examined under a light microscope (ECLIPSE E200; Nikon Corporation, Tokyo, Japan) to assess tissue integrity, cellular organization, and structural alterations in the stem tissues.

An additional experiment was conducted to investigate symptom development following stem-base wounding in the absence of *S. rolfsii* SCO1. The experiment included a wounded mock-inoculated control (S-Tr1) and THR3-treated seedlings (S-Tr2). The experimental procedures and disease assessment criteria are described in Supplementary Method S1.

### 4.7. Statistical Analysis

Disease incidence (DI) was calculated as the percentage of symptomatic plants within each treatment, whereas disease severity (DS) was determined using a 0–4 disease rating scale. For the in planta biocontrol experiment, DI was recorded as a binary outcome at the individual-plant level (0 = asymptomatic; 1 = symptomatic). Differences in DI among treatments at each assessment time were evaluated using the Fisher–Freeman–Halton exact test. Disease severity was expressed as mean ± standard deviation (SD) and analyzed using one-way analysis of variance (ANOVA) at each assessment time.

For the longitudinal evaluation of the in planta biocontrol experiment, exploratory permutation-based analyses were performed using Python (version 3.13.5; Python Software Foundation; https://www.python.org/) and NumPy (version 2.3.5; https://numpy.org/), with individual plants treated as repeated observational units. The effects of treatment, assessment time, and their interaction were evaluated using sum-of-squares (SS) statistics. Treatment effects were examined by randomly reallocating complete plant-level longitudinal trajectories among the four treatment groups while maintaining five plants per group. Assessment-time effects were explored by permuting time labels within individual plants, whereas treatment × time interactions were assessed by permuting plant-mean-centered longitudinal trajectories among treatment groups. Monte Carlo *p*-values were estimated from 200,000 permutations using the PCG64 random-number generator with a fixed random seed of 20261028. Each *p*-value was calculated as (b + 1)/(B + 1), where b denotes the number of permuted test statistics equal to or greater than the observed statistic, and B represents the total number of permutations. The original *p*-values were not adjusted for multiple testing. Holm-adjusted *p*-values were subsequently calculated across the six omnibus tests as a sensitivity analysis. The corresponding exploratory test statistics and unadjusted *p*-values are presented in Table 4. Because the validity of the time-label and centered-trajectory permutation procedures depends on exchangeability assumptions that have not been fully verified for progressive disease measurements, these longitudinal results were considered exploratory rather than confirmatory.

For pathogenicity and dual-culture assays, treatment comparisons were performed using ANOVA. Where appropriate, mean comparisons were conducted using Fisher’s least significant difference (LSD) test at *p* < 0.05. Conventional statistical analyses were performed using IBM SPSS Statistics (version 31; IBM Corp., Armonk, NY, USA). Statistical significance was assessed at a nominal threshold of *p* < 0.05.

## 5. Conclusions

Among the three *Sclerotium rolfsii* isolates evaluated, SCO1 showed the greatest aggressiveness on two-month-old durian seedlings. *Trichoderma* sp. THR3 inhibited SCO1 mycelial growth by 88.7% in the dual-culture assay and was associated with a 50% numerical reduction in final disease severity relative to the pathogen-inoculated control in one-year-old grafted durian plants. These findings suggest that THR3 is a promising candidate for biological control of *S. rolfsii* SCO1 under the experimental conditions evaluated. However, the in planta assessment was limited to one pathogen isolate, one biocontrol strain, and a single pot experiment without independent repetition or comparison with chemical fungicides and reference biocontrol strains. Further investigations, building on the supplementary wounded-control experiment, are needed to clarify the causes of symptoms observed following THR3 application and to establish the identity and potential role of microorganisms recovered from symptomatic tissues.

## Figures and Tables

**Table 1:** Pathogenicity test of *Sclerotium rolfsii* (SCO1, SCO2, and SCO3) on two-month-old durian seedlings.

Treatment	Disease Incidence (DI) (%)				Disease Severity (DS) (%)			
Seedling Stage (2 Months)	3 DAI	5 DAI	7 DAI	9 DAI	3 DAI	5 DAI	7 DAI	9 DAI
Healthy control	0.0 ± 0.00 ^c^	0.0 ± 0.00 ^c^	0.0 ± 0.00 ^c^	0.0 ± 0.00 ^c^	0.0 ± 0.00 ^c^	0.0 ± 0.00 ^c^	0.0 ± 0.00 ^c^	0.0 ± 0.00 ^c^
*S. rolfsii* SCO1	100.0 ± 0.00 ^a^	100.0 ± 0.00 ^a^	100.0 ± 0.00 ^a^	100.0 ± 0.00 ^a^	85.0 ± 12.91 ^a^	92.5 ± 12.8 ^a^	100.0 ± 0.00 ^a^	100.0 ± 0.00 ^a^
*S. rolfsii* SCO2	60.0 ± 51.64 ^b^	60.0 ± 51.64 ^b^	80.0 ± 42.16 ^ab^	80.0 ± 42.16 ^b^	35.2 ± 31.29 ^b^	35.2 ± 31.29 ^b^	52.5 ± 29.93 ^ab^	80.0 ± 42.16 ^b^
*S. rolfsii* SCO3	40.0 ± 51.64 ^b^	40.0 ± 51.64 ^b^	60.0 ± 51.64 ^b^	80.0 ± 42.16 ^b^	25.0 ± 33.33 ^b^	47.5 ± 42.57 ^b^	47.5 ± 43.22 ^b^	52.5 ± 29.93 ^b^
F-value	13.00	13.00	16.80	22.13	14.206	12.255	9.113	6.382
*p*-value	<0.001	<0.001	<0.001	<0.001	<0.001	<0.001	<0.001	<0.001
LSD (0.05)	33.12	33.12	30.23	27.04	25.17	28.71	27.85	27.39
C.V. (%)	73.03	73.03	55.56	45.87	57.74	59.60	45.53	38.52

*Notes:* Values are expressed as mean ± standard deviation (SD) from 10 biological replicates (*n* = 10), with one seedling per replicate. DAI = days after inoculation. Disease incidence (DI, %) was calculated as the number of symptomatic seedlings divided by the total number of seedlings assessed × 100. Disease severity (DS, %) was calculated using a disease rating scale of 0–4, where 0 represents no disease symptoms and 4 represents maximum disease severity. Different lowercase letters within the same column indicate significant differences among treatments according to Fisher’s least significant difference (LSD) test at *p* < 0.05. C.V. = coefficient of variation.

**Table 2:** In vitro antagonistic activity of *Trichoderma* sp. THR3 against three isolates of *Sclerotium rolfsii* in dual-culture assays.

Pathogens	*Trichoderma* sp. THR3								
	Control Diameter (cm)			Dual-Culture Diameter (cm)			Mycelial Growth Inhibition (%)		
	3 DAI	5 DAI	7 DAI	3 DAI	5 DAI	7 DAI	3 DAI	5 DAI	7 DAI
*S. rolfsii* SCO1	2.8 ± 0.06 ^b^	4 ± 0.06 ^c^	9 ± 0.00	0.90 ± 0.03 ^c^	0.98 ± 0.03 ^c^	1.01 ± 0.03 ^c^	67.7 ± 0.87 ^a^	75.4 ± 0.59 ^a^	88.7 ± 0.30 ^a^
*S. rolfsii* SCO2	3 ± 0.06 ^b^	5 ± 0.06 ^b^	9 ± 0.00	1.60 ± 0.03 ^b^	1.73 ± 0.03 ^b^	1.81 ± 0.03 ^b^	46.5 ± 1.02 ^b^	65.4 ± 0.49 ^b^	79.8 ± 0.30 ^b^
*S. rolfsii* SCO3	6 ± 0.06 ^a^	8 ± 0.06 ^a^	9 ± 0.00	5.26 ± 0.03 ^a^	5.74 ± 0.03 ^a^	5.80 ± 0.03 ^a^	12.3 ± 0.80 ^c^	28.2 ± 0.49 ^c^	35.5 ± 0.30 ^c^
F-value	4016.67	5416.67	n/a	37,810.57	45,253.32	45,407.13	4842.25	11,201.99	45,407.13
*p*-value	<0.001	<0.001	n/a	<0.001	<0.001	<0.001	<0.001	<0.001	<0.001
LSD (0.05)	0.0872	0.0872	n/a	0.0371	0.0371	0.0371	1.2407	0.7252	0.4123
C.V. (%)	1.61	1.12	0.00	1.04	0.96	0.94	2.13	0.93	0.44

*Notes*: Values are presented as mean ± standard deviation (SD) of five replicates (*n* = 5). Means followed by different lowercase letters within the same column indicate significant differences among *Sclerotium rolfsii* isolates according to Fisher’s protected least significant difference (LSD) test at *p* < 0.05. Mycelial growth inhibition (%) was calculated using the formula [(C1 − C2)/C1] × 100, where C1 represents the colony diameter of *S. rolfsii* in the control treatment and C2 represents the colony diameter of *S. rolfsii* in dual culture with *Trichoderma* sp. THR3. DAI, days after inoculation; C.V., coefficient of variation; LSD, least significant difference; n/a, not applicable.

**Table 3:** Efficacy of *Trichoderma* sp. THR3 in controlling *Sclerotium rolfsii* (SCO1) causing damping-off disease in one-year-old grafted durian plants.

Treatment	Disease Incidence (Number of Diseased Plants/Total Plants)				Disease Severity (DS %)				Disease Reduction Percentage (DRP) (%)
	3 DAI	5 DAI	7 DAI	9 DAI	3 DAI	5 DAI	7 DAI	9 DAI	9 DAI
Tr1 (Healthy control)	0 (0/5)	0 (0/5)	0 (0/5)	0 (0/5)	0 ± 0	0 ± 0	0 ± 0	0 ± 0	n.a.
Tr2 (THR3)	20 (1/5)	20 (1/5)	20 (1/5)	20 (1/5)	10 ± 22.36	20 ± 44.72	20 ± 44.72	20 ± 44.72	n.a.
Tr3 (SCO1)	60 (3/5)	80 (4/5)	100 (5/5)	100 (5/5)	30 ± 27.39	50 ± 35.36	65 ± 37.91	100 ± 0	0
Tr4 (SCO1 + THR3)	40 (2/5)	60 (3/5)	60 (3/5)	60 (3/5)	25 ± 35.36	35 ± 37.91	45 ± 51.23	50 ± 50.00	50
F-value	-	-	-	-	1.517	1.947	2.667	8.407	-
*p*-value	0.3808	0.0633	0.0075	0.0075	0.2484	0.1628	0.0829	0.0014	-
Significance	ns	ns	**	**	ns	ns	ns	**	-
LSD (0.05)	-	-	-	-	33.52	45.90	52.20	44.97	-
C.V. (%)	-	-	-	-	153.85	130.41	119.79	78.92	-

*Note:* Disease incidence (DI) is expressed as the percentage of diseased plants, with the number of diseased plants relative to the total number of plants indicated in parentheses (*n* = 5 per treatment). Disease severity (DS) is presented as mean ± standard deviation (SD) based on five biological replicates, with one plant per replicate. Differences in DI among treatments at each assessment time were evaluated using the Fisher–Freeman–Halton exact test, whereas DS was analyzed using one-way analysis of variance (ANOVA). Fisher’s least significant difference (LSD) values at the 5% significance level are presented for reference; pairwise significance groupings are not displayed. Disease reduction percentage (DRP) at 9 days after inoculation (DAI) was calculated as [(DS of Tr3 − DS of Tr4)/DS of Tr3] × 100, using the pathogen-inoculated control (Tr3) as the reference. DRP was not calculated for Tr1 and Tr2 because these treatments were not inoculated with *Sclerotium rolfsii* SCO1. The statistical analyses presented in this table represent comparisons among treatments at individual assessment times, whereas longitudinal effects are presented separately in Table 4. DI, disease incidence; DS, disease severity; DRP, disease reduction percentage; DAI, days after inoculation; LSD, least significant difference; C.V., coefficient of variation; n.a., not applicable; ns, not significant (*p* ≥ 0.05); **, significant at *p* < 0.01.

**Table 4:** Longitudinal permutation analysis of disease incidence (DI) and disease severity (DS) in one-year-old grafted durian plants.

Source of Variation	DI Test Statistic (SS)	DI *p*-Value	DS Test Statistic (SS)	DS *p*-Value
Treatment (T)	2.1250	0.0147	16.8625	0.0446
Time (D)	0.3000	0.1248	11.6500	<0.001
T × D	0.4000	0.5603	12.8500	0.0008

Note: DI was recorded as a binary outcome, whereas DS was evaluated using a 0–4 disease rating scale. Exploratory longitudinal permutation analyses were conducted using plant-level repeated observations. SS represents the sum-of-squares test statistic. Monte Carlo *p*-values were calculated using 200,000 permutations and are presented without adjustment for multiple testing.

## Data Availability

The data supporting the findings of this study are available from the corresponding author upon reasonable request.

## Supplementary Materials

The following supporting information can be downloaded at: https://www.mdpi.com/article/10.3390/plants15213454/s1, Table S1. Disease incidence and disease severity of durian seedlings subjected to stem-base wounding and sterile PDA disc placement, with or without *Trichoderma asperellum* THR3; Figure S1. Representative stem-base symptoms and fungal observations from the supplementary wounded mock-inoculation experiment; Figure S2. ITS sequence identification report of *Trichoderma* sp. THR3 obtained from Macrogen, showing 100% sequence identity (602/602 bp) with a GenBank sequence annotated as *Trichoderma asperellum* (accession number KM456214.1).
