# Supplementary material for: Biocontrol Efficacy of Trichoderma sp. THR3 Against Sclerotium rolfsii SCO1 Causing Damping-Off in Durian Seedlings
Source: Plants (Basel). 2026 Nov 5;15(21):3454. doi: 10.3390/plants15213454 (PMC13766065; doi:10.3390/plants15213454)
Supplement: Supplementary file 1 [file plants-15-03454-s001.zip › plants-4614340-supplementary.pdf]

Supplementary Materials

Supplementary Evaluation of Stem-Base Symptoms in Wounded Durian Seedlings with and Without *Trichoderma asperellum* THR3 Application

S1. Supplementary Materials and Methods

An additional experiment was conducted to investigate the development of stem-base symptoms in durian seedlings following wounding, with or without the application of *Trichoderma asperellum* THR3. The experimental procedures and nursery conditions were consistent with those described for the main biocontrol experiment in Section 4.6, except that only two treatments were evaluated. The treatments were as follows: S-Tr1 (Wounded mock-inoculated control): Seedlings were wounded at the stem base, and sterile potato dextrose agar (PDA) discs without fungal inoculum were placed at the wound sites 72 h after wounding. S-Tr2 (THR3-treated seedlings): Seedlings were wounded at the stem base, and PDA discs colonized by *T. asperellum* THR3 were placed at the wound sites 72 h after wounding. Neither treatment was inoculated with *Sclerotium rolfsii* SCO1. Each treatment consisted of five durian seedlings. Disease incidence (DI) and disease severity (DS) were assessed at 3, 5, 7, and 9 days after inoculation (DAI), with the day of PDA disc placement designated as 0 DAI. The same disease assessment criteria and calculation methods as those employed in the main experiment were used.

S2. Supplementary Results

Stem-base symptoms were observed in both the wounded mock-inoculated control (S-Tr1) and THR3-treated seedlings (S-Tr2). In S-Tr1, disease incidence increased from 40% at 3 DAI to 60% at 5 DAI and remained unchanged through 9 DAI, whereas disease severity increased from 10% to 30%.

In S-Tr2, disease incidence remained at 20% throughout the assessment period, while disease severity increased from 5% at 3 DAI to 15% at 9 DAI (Table S1). These observations demonstrate that stem-base symptoms developed in wounded seedlings even without deliberate fungal inoculation. However, the cause of the symptoms was not established, and the results do not independently confirm the pathogenicity or protective activity of THR3.

Table S1. Disease incidence and disease severity of durian seedlings subjected to stem-base wounding and sterile PDA disc placement, with or without *Trichoderma asperellum* THR3.

Treatment	DAI	Number of seedlings	Symptomatic seedlings	DI (%)	DS (%)
S-Tr1 (Wounded mock-inoculated control)	3	5	2	40 00 ± 54.77	10 ± 13.69
	5	5	3	60 ± 54.77	25 ± 22.36
	7	5	3	60 ± 54.77	30 ± 35.36
	9	5	3	60 ± 54.77	30 ± 41.08
S-Tr2 (THR3-treated seedlings)	3	5	1	20 ± 44.72	5 ± 11.18
	5	5	1	20 ± 44.72	5 ± 22.36
	7	5	1	20 ± 44.72	10 ± 33.54
	9	5	1	20 ± 44.72	15 ± 44.72
F-value	9	n/a	n/a	0.400	0.136
p-value	9	n/a	n/a	0.545	0.722
LSD (0.05)	9	n/a	n/a	72.92	62.62
C.V. (%)	9	n/a	n/a	66.67	71.76

Notes: Values are presented as mean ± standard deviation (SD) from five biological replicates (n = 5), with one durian seedling per replicate. DAI = days after inoculation or treatment; DI = disease incidence; DS = disease severity; LSD = least significant difference; and C.V. = coefficient of variation. Disease incidence was calculated as the percentage of seedlings exhibiting disease symptoms, whereas disease severity was determined using a 0–4 disease rating scale and expressed as a percentage. F-

values and p-values were obtained from one-way analysis of variance (ANOVA), and LSD values were calculated at the 5% significance level. No significant differences were detected between treatments at any assessment time ($p > 0.05$). S-Tr1 and S-Tr2 refer exclusively to the supplementary experiment and are distinct from the treatment numbering used in the main experiment.

Representative photographs of durian seedlings subjected to stem-base wounding followed by placement of sterile PDA discs (S-Tr1) or PDA discs colonized by *Trichoderma asperellum* THR3 (S-Tr2). Both treatments were applied 72 h after wounding. Images show the external symptoms and associated fungal observations from the supplementary experiment. (Figure S1)

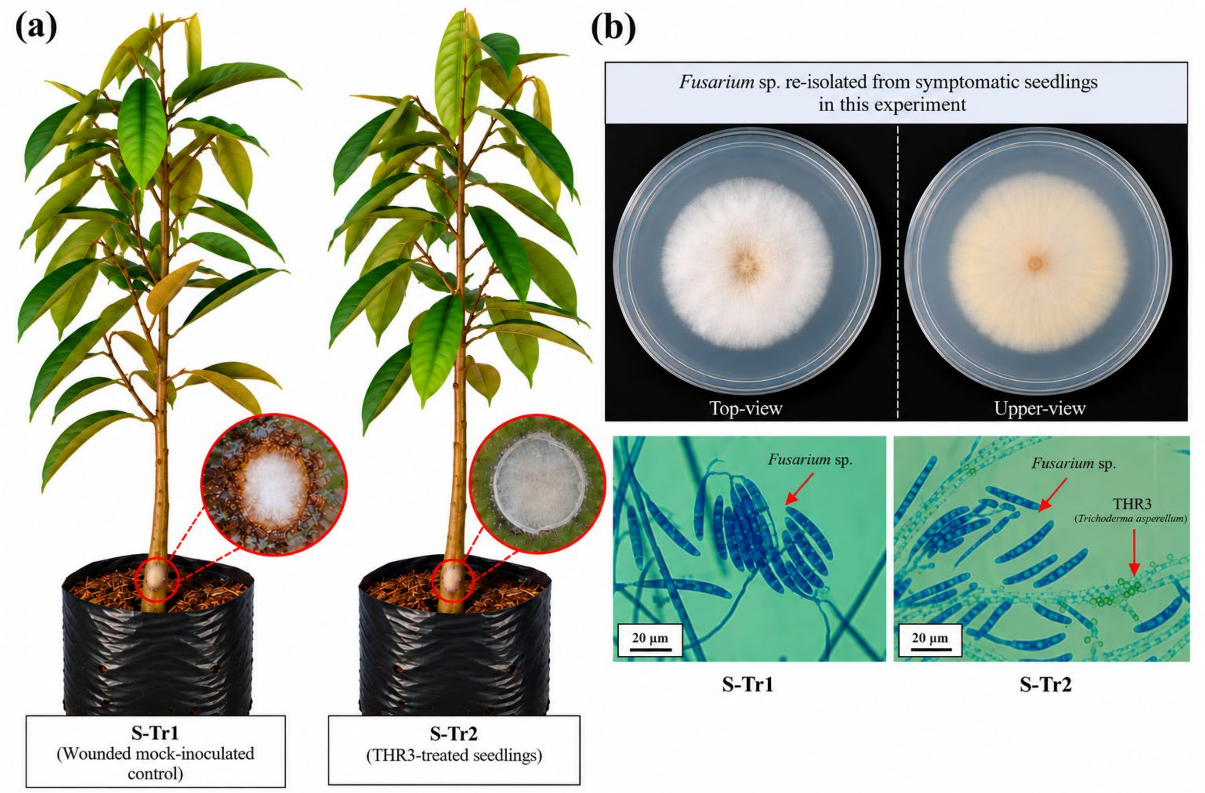

Figure S1. Representative stem-base symptoms and fungal observations from the supplementary wounded mock-inoculation experiment.

Standard ID

ITS Region Sequencing | Humanizing Genomics macrogen

ITS region service report

Order Number : HC00629973
Sample name : THR3_contig_1

Information

Primer Information

Sequencing Primer Name Primer Sequences	PCR Primer Name Primer Sequences
ITS5 5' (TCC GTA GGT GAA CCT GCG G) 3'	ITS5 5' (TCC GTA GGT GAA CCT GCG G) 3'
ITS4 5' (TCC TCC GCT TAT TGA TAT GC) 3'	ITS4 5' (TCC TCC GCT TAT TGA TAT GC) 3'

Subject						Score		Identities	
Accession	Description	Length	Start	End	Coverage	Bit	E-Value	Match/Total	Pct.(%)
KM456214.1	Trichoderma asperellum	645	633	32	-93	1112	0.0	602/602	100

Kingdom	Family	Genus	Species
Eukaryota	Hypocreaceae	Trichoderma	Trichoderma asperellum

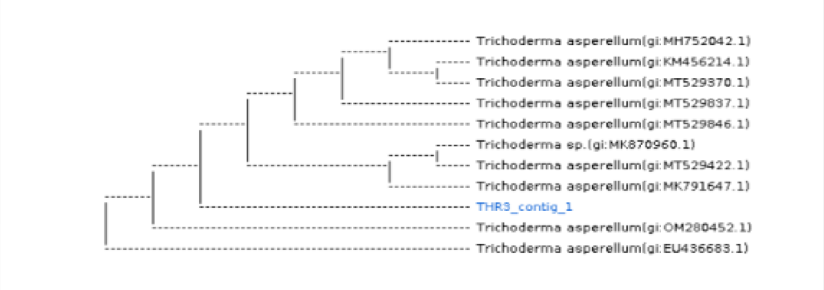

Characterization

Figure S2. ITS sequence identification report of *Trichoderma* sp. THR3 obtained from Macrogen, showing 100% sequence identity (602/602 bp) with a GenBank sequence annotated as *Trichoderma asperellum* (accession number KM456214.1).
